# Evaluation of the Ability of Nanostructured PEI-Coated Iron Oxide Nanoparticles to Incorporate Cisplatin during Synthesis

**DOI:** 10.3390/nano7100314

**Published:** 2017-10-12

**Authors:** Raluca Tutuianu, Laura Madalina Popescu, Mihai Bogdan Preda, Ana-Maria Rosca, Roxana Mioara Piticescu, Alexandrina Burlacu

**Affiliations:** 1 Institute of Cellular Biology and Pathology “NicolaeSimionescu”, Laboratory of Stem Cell Biology, 8 B.P. Hasdeu Street, 050568 Bucharest, Romania; raluca.tutuianu@icbp.ro (R.T.); bogdan.preda@icbp.ro (M.B.P.); ana-maria.rosca@icbp.ro (A.-M.R.); 2 National R&D Institute for Non-Ferrous and Rare Metals, 102 Biruintei Blvd, 077145 Pantelimon, Romania; mpopescu@imnr.ro (L.M.P.); roxana.piticescu@imnr.ro (R.M.P.)

**Keywords:** poly(ethileneimine) iron oxide hybrid nanoparticles, hydrothermal synthesis, drug release, mesenchymal stem cells, tumor cells

## Abstract

Nanoparticles (NPs) have a high potential for biological applications as they can be used as carriers for the controlled release of bioactive factors. Here we focused on poly(ethylenimine) (PEI)-coated iron oxide hybrid NPs obtained by hydrothermal synthesis in high pressure conditions and evaluated their behavior in culture medium in the presence or absence of cells, as well as their ability to incorporate antitumor drug cisplatin. Our results showed that the hydrothermal conditions used for Fe-PEI NPs synthesis allowed the incorporation of cisplatin, which even increased its anti-tumor effects. Furthermore, the commonly occurring phenomenon of NPs aggregation in culture medium was exploited for further entrapment of other active molecules, such as the fluorescent dye DiI and valinomycin. The molecules bound to NPs during synthesis or during aggregation process were delivered inside various cells after in vitro and in vivo direct contact between cells and NPs and their biological activity was preserved, thus supporting the therapeutic value of Fe-PEI NPs as drug delivery tools.

## 1. Introduction

Nanoparticles (NPs) have a great potential for biological applications [1,2,3] and, among them, dendrimer-based iron oxide NPs occupy a prominent place owing to their ability to work both as MRI contrast agents [1,2] and drug delivery systems [3]. In addition to magnetic properties, iron oxide NPs are valued for their biocompatibility, injectability, lack of toxicity, and high-level accumulation in target tissues [4,5], therefore being proposed as agents for magnetic resonance imaging, localized hyperthermia treatment, controlled drug release, magnetic guidance and manipulation [6]. Superparamagnetic iron oxide NPs (SPIONs) were initially considered as the ideal magnetic NPs due to their small tendency to agglomeration [7,8]. However, it turned out shortly that naked SPIONs, namely NPs without a polymeric coating, did show not only aggregation in water, but also chemical instability in air [9]. Moreover, SPIONs generally have a relatively low saturation magnetization and coercivity and a diminished heating efficiency, which all limit their applicability in magnetic hyperthermia [10]. Coating the surface of SPIONs with different compounds, such as surfactants, silica or various hydrophilic, biocompatible polymers increased their stability in aqueous media, as well as their functionalization, biocompatibility and cellular uptake [5,9]. Among polymers, several classes of polysaccharides (such as starch, agarose, alginate, chitosan, dextran, pullulan, and heparin) have been used to coat SPIONs. Chitosan-based Fe_3_O_4_-Curcumin conjugate has been reported to behave as a multifunctional nanosystem, having antibacterial and antiproliferative activities and drug delivery properties [11]. Functional magnetic nanoconjugates composed of SPIONs coated by alginate and curcumin were also engineered and validated for anti-cancer drug delivery and hyperthermia [12]. In addition, Lin et al. [13] have developed a novel type of nanometer-sized SPIONs complexed with amylose and cationized with spermine (ASP-SPIONs), which were delivered into mesenchymal stem cells (MSC) and used for in vivo MRI tracking after transplantation.

One of the great advantages brought by dendrimer-based NPs in comparison to traditional therapeutics is their superior capability to deliver poorly water-soluble active substances while ensuring a high intracellular dose in the targeted cells [14,15]. However, dendrimer-based NPs do not behave in solution as inert or soluble small molecules. Rather, the active surface chemistry of their coating generates multiple interactions with the environment components, in an attempt to minimize the surface energy. In biomedical applications, their dispersion in the cell culture medium is often followed by NPs aggregation into large clusters, leading to the formation of new molecular entities. Furthermore, it has become apparent that a commonly occurring phenomenon upon dispersion of NPs in the culture media is the protein corona formation giving rise to increased-sized NPs (or aggregates), which are actually what cells see and interact with [16,17,18].

One of the most attractive cells with high impact in biomedical applications and particularly in the field of regenerative medicine are the mesenchymal stem cells (MSCs), which are currently being evaluated in more than 500 clinical trials (https://clinicaltrails.gov) for the treatment of a broad range of disorders, including cancer and degenerative diseases. These cells exert their beneficial effects on tissue repair by either paracrine actions or direct differentiation. The active secretion of bioactive molecules and modulation of the local immune response are the main mechanisms by which these cells locally produce a regenerative microenvironment after transplantation, thus serving as site-regulated “drug stores” in vivo [19]. Loading stem cells with magnetic NPs before transplantation provides a mean to track the migration of these cells to sites of disease. Moreover, the magnetic NPs may be specially designed so that to deliver various drugs or other biomolecules at the site of transplantation, which might improve the regenerative properties of transplanted cells.

However, understanding the mechanisms underlying the interaction of NPs with specific biological environment (cells, serum components, biomolecules of the culture medium, surface coatings, etc.) is a prerequisite before proceeding into more in depth studies on the effectiveness of magnetic NPs-based vector systems for drug delivery.

In our previous work, hydrothermal procedures (at 1000–3000 atm and 40 °C) were used to prepare hybrid nanomaterials based on iron oxide NPs and branched polyethyleneimine (PEI) with different iron oxide: PEI mass ratios (1.5, 1 and 0.5). FeO(OH) crystallites with dimensions of 2–5 nm were formed in all these conditions. Furthermore, we found that synthesis of PEI-coated iron oxyhydroxide was favored when lower pressure (1000 atm) and an iron oxide:PEI mass ratio of 1:2 wasused. On the other hand, we had previously reported that magnetite NPs (Fe_3_O_4_) were formed in hydrothermal conditions at 100 atm [20]. PEI-coated Fe_3_O_4_, in which PEI was used as a stabilizer to form iron oxide NPs with a size range of 16–22 nm, was also synthesized using hydrothermal approach at 134 °C with a gauge pressure of 2 bar [21]. These NPs showed good hemocompatibility and cytocompatibility after surface modification with PEG moieties, acetyl groups, or carboxyl groups, and become valuable in magnetic resonance imaging and therapy [22]. Based on these findings, in the present study we focused on the poly(ethylenimine) (PEI)-coated iron oxide hybrid NPs with 2–5 nm in diameter, which were obtained by hydrothermal synthesis at 100 atm and 40 °C, starting from an iron oxide:PEI mass ratio of 1:2. Our work evaluated the behavior of these hybrid nanostructures in the culture medium in the presence and absence of various cells, as well as their ability to incorporate biologically active factors, such as fluorescent molecule DiI, valinomycin and cisplatin.

## 2. Results

### 2.1. Selection and Characterization of Fe-PEI NPs

**Figure A1:**
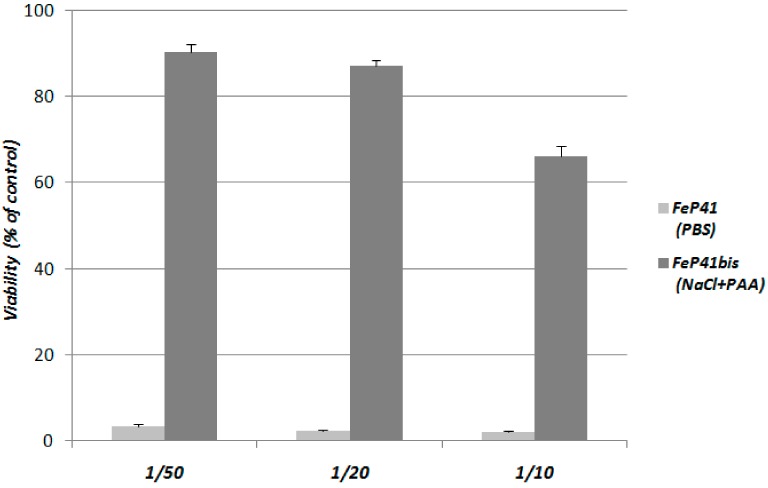
Viability of Luc-expressing U87 cells in the presence of Fe-PEI NPs prepared in PBS (FeP 41) and PAA/NaCl solution (FeP41 bis). NPs were diluted in complete culture medium at various dilution, i.e., 1/50, 1/20, and 1/10. Note that the bioluminescent signal produced by Luc-expressing cells after interaction with NPs revealed a cytotoxic effect of samples prepared in PBS as dispersing medium on tumor cells, in comparison to similar samples prepared in PAA-based dispersion medium.

The optimal synthesis conditions were established by analyzing the behavior of various NPs in culture medium, in the absence or presence of a variety of cell types (endothelial cells, macrophage cell line RAW 264.7 from ATCC, mesenchymal stem cells, brain tumor cells). To this aim, several synthesis conditions were evaluated, which varied in the initial Fe:PEI mass ratio (1:2, 1:1, 3:2), hydrothermal pressure conditions (100 vs. 1000 atm, 1, 3 or 5 h) and dispersing medium (PBS vs. PAA). The selection criteria were based on the lack of cytotoxic effect on cells in culture, the stability of NPs in culture medium, as well as the formation of NPs aggregates upon interaction with cultured cells. This preliminary work has led to the elimination of several synthesis conditions, such as resuspension of Fe-PEI lyophilizate in PBS, or assembling at high pressure conditions, as they led to cytotoxic and instable NPs (Figure A1 and data not showed). Finally, the following NPs synthesis conditions were retained for further investigations: initial Fe:PEI mass ratio of 1:2, one-hour hydrothermal synthesis at 100 atm hydrothermal pressure and 40 °C, and resuspension of the lyophilizate in sodium chloride solution in the presence of PAA for stabilization.

The selected NPs were further characterized by FT-IR (Fourier Transformed Infrared Spectroscopy) and DSC-TG (Differential Scanning Calorimetry and Thermogravimetry) analysis, particle size measurement and HRTEM (High-Resolution Transmission Electron Microscopy). The magnetic characterization of the iron oxide NPs and Fe-PEI NPs has been previously published elsewhere [22].

**Figure 1:**
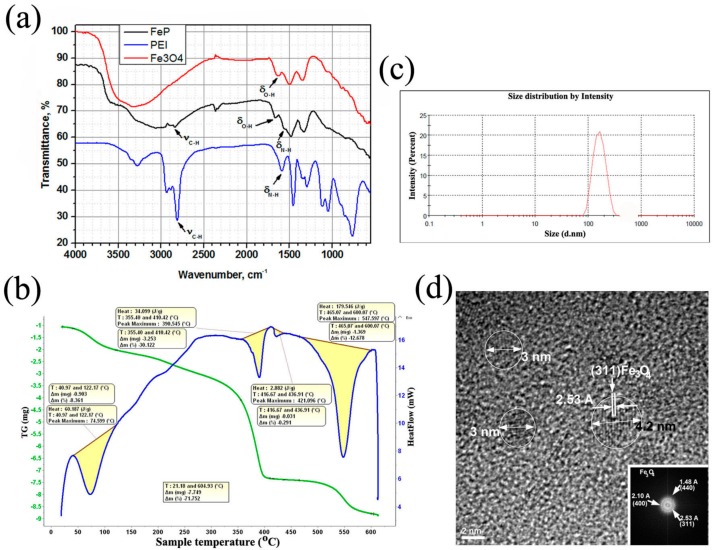
Characterization of Fe-PEI NPs. (**a**) FT-IR spectra of PEI and Fe-PEI nanohybrids; (**b**) DSC-TG thermograms of Fe-PEI showing three main endothermic peaks; (**c**) Particle size distribution of Fe-PEI hybrids; (**d**) HRTEM micrograph of Fe-PEI-based nanostructured hybrids. Inset illustrates interplanar distances corresponding to Miller indexes of magnetite NPs.

FT-IR spectrum of a sample of Fe-PEI NPs is presented in Figure 1a, in comparison to PEI and iron oxide NPs alone. Characteristic peaks of PEI, such as stretching vibration of C–H (2810 cm^−1^) and bending vibration of N–H group (1586 cm^−1^), were found in nanostructured hybrid, slightly shifted at 2834 cm^−1^ (ν C–H) and 1546 cm^−1^ (δ N–H), respectively. Also, characteristic peaks of iron oxide NPs such as H_2_O bending vibration (1626 cm^−1^), Fe–O–H bending vibration (890 cm^−1^) and specific stretching vibration of Fe–O (595 cm^−1^) were observed in Fe-PEI hybrid at 1658 cm^−1^ (δ H_2_O), 842 cm^−1^ (δ Fe–O–H) and at 562 cm^−1^ (ν Fe–O), respectively. Stretching vibration of water adsorbed molecules was shifted from 3302–3420 cm^−1^ in pure iron oxide to 3166–3062 cm^−1^ in Fe-PEI nanopowders (Figure 1a).

DSC-TGthermogram of hybrid nanostructures (Figure 1b) displayed three main endothermic peaks. The first peak at 74–78 °C corresponded to the loss of humidity. Elimination of physically adsorbed water at temperatures below 100 °C could be explained by the formation of physical bonds inside inorganic-organic hybrids, as previously described [9]. The second peak, between 390–420 °C could be attributed to PEI degradation [23,24]. The weight loss associated with this peak was ~20–30%, representing the decomposition of PEI component. The lower decomposition temperature of PEI in this case, compared to pure PEI (500–600 °C) was probably due to hybrid nanostructure. The third peak at 547 °C could be assigned to free residual PEI decomposition (Figure 1b).

The mean particle size of Fe-PEI hybrids measured in sterile filtered aqueous suspensions was 154.4 nm with a polydispersity index of 0.074 (Figure 1c).

The HRTEM image of Fe-PEI hybrid showed small crystalline inorganic particles. NPs sizes were in the range of 2–5 nm. Interplanar distances were calculated (2.53 Å) and found to correspond to Miller indexes of magnetite Fe_3_O_4_ NPs [25]. Nanocrystallinity of iron oxide NPs was demonstrated by characteristic SAED (Selected Area Electron Diffraction) pattern of Fe_3_O_4_ (Figure 1d).

### 2.2. Aggregation of Fe-PEI NPs in the Culture Medium

**Figure A2:**
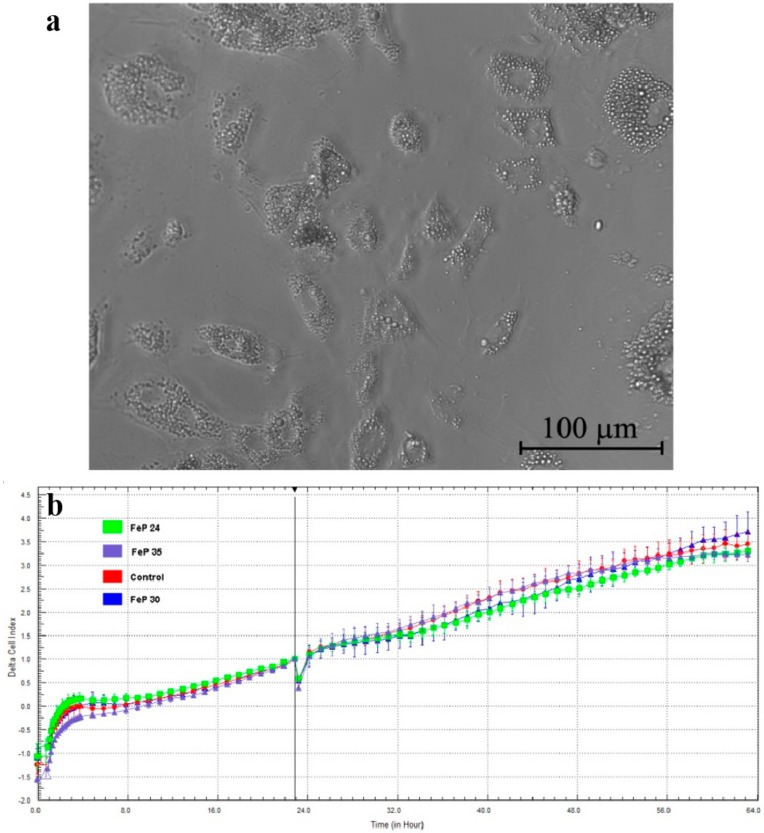
Biocompatibility of Fe-PEI NPs. (**a**) Phase-contrast microscopy illustrating the healthy aspect of endothelial progenitor cells (EPCs) after interaction with NPs. Note the specific accumulation of NPs as aggregates onto cell surface. (**b**) XCELLigence analysis illustrating that addition of NPs to cell culture does not affect cell index (a measure of cell morphology, cell adherence to substrate and cell proliferation rate). Three different NPs were used for this analysis, which were all obtained in the same synthesis conditions (initial mass ratio Fe:PEI = 1:2, 100 atm, 1 h, 40 °C, stabilization in PAA-saline solution, see main text for details).

Biocompatibility of Fe-PEI NPs was first evaluated by interaction with various cell types, including MSCs and EPCs. As illustrated in Figure A2 and Supplemental Movie 1, cell proliferation was not affected by the presence of NPs and Fe-PEI NPs rapidly accumulated onto the cell surface and assembled into micron-level aggregates upon interaction with cells.

**Figure 2:**
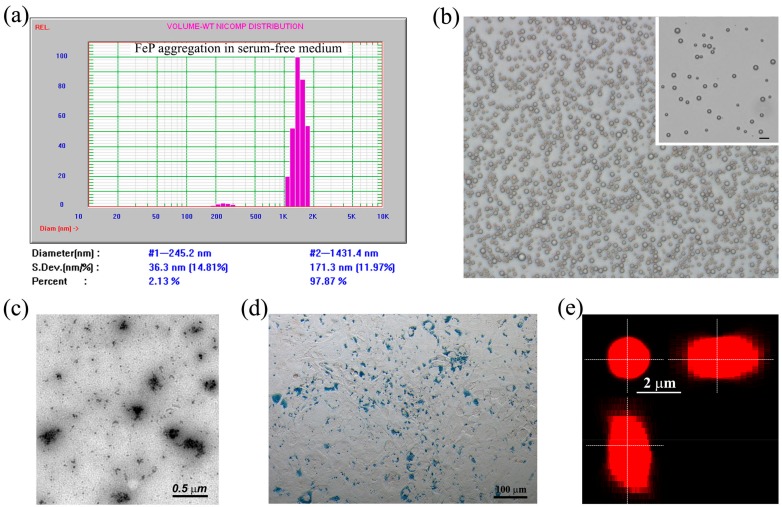
Characterization of Fe-PEI aggregates formed by 24-h incubation in culture medium. (**a**) Nicomp particle size distribution of Fe-PEI aggregates shaped by 24-h of NPs culture in DMEM. The diagrams are representative of at least three experiments performed in duplicate. (**b**)Phase-contrast image illustrating the aggregates formed by incubation of Fe-PEI NPs in DMEM in ultra-low attachment conditions. The inset shows a diluted aggregate suspension (scale bar: 5 µm) illustrating that the aggregates are individual rather than being arranged in strings; (**c**) Electron microscopy micrograph illustrating Fe-PEI aggregates; (**d**) Light microscopy image illustrating the appearance of blue aggregates on MSCs, which are indicative of ferric iron accumulation onto cell surface, after 24-h co-culture with Fe-PEI NPs; (**e**) Orthogonal confocal optical sections of a fluorescently labelled Fe-PEI aggregate produced by incorporation of DiI during assembling demonstrate the uniform distribution of fluorescence within aggregate.

**Figure A3:**
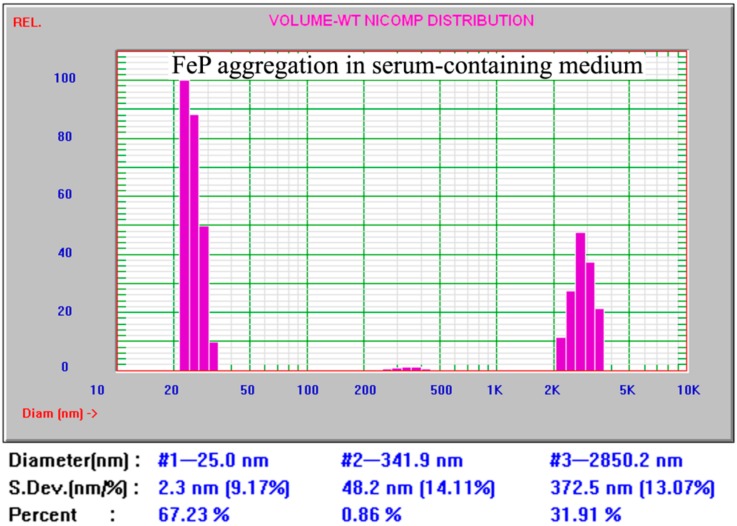
Nicomp particle size distribution of Fe-PEI aggregates shaped by 24 h culture in DMEM (Dulbecco’s Modified Eagle Medium) in the presence of 10% FCS. Note that a smaller number of NPs are assembled into aggregates of 2.85 ± 0.37 µm, larger than those obtained in serum-free medium.

Incubation of Fe-PEI NPs in culture medium, in the absence of cells gave rise to aggregates of 1431 ± 171 nm (Figure 2a). NPs aggregation was independent of the presence of fetal calf serum (FCS) in the culture medium; however, aggregation in the presence of 10% FCS in DMEM was more chaotic (Figure A3), as many Fe-PEI NPs remained non-aggregated after 24 h.

These aggregates were also characterized by phase-contrast microscopy (Figure 2b) and transmission electron microscopy (Figure 2c), which both revealed the presence of aggregates with sizes ranging from 200 nm to 2 µm. These aggregates stained positively for ferric iron, as revealed by Prussian Blue staining of MSCs after 24-h of culture in the presence of Fe-PEI NPs (Figure 2d). It is worth noting that aggregation of Fe-PEI NPs was produced in culture medium both in tissue culture-treated plates (in conditions favoring cell adhesion), as well as in ultra-low adherence (ULA) plates (in conditions not-favoring cell adhesion), and in both cases adhesive aggregates were produced. This aspect is important because we used these properties of post-synthesis aggregation in both conditions for further entrapment of various biologically active molecules. First, DiI fluorescent molecule was used to assess the possibility of entrapment of exogenous molecules in Fe-PEI aggregates. The incorporation of DiI was effective (more than 90% efficiency) and uniform, as demonstrated by spectrofluorometric measurement of the fluorescence (using excitation with white light and emission wavelength 610 ± 10 nm) in culture medium, as well as by confocal microscopy on 2.5 µm Z-stack (Figure 2e). These data indicated that the aggregation of Fe-PEI NPs in the culture medium could be exploited to incorporate exogenous molecules present in the medium.

### 2.3. Fe-PEI NPs Incorporate Fluorescent Tracer DiI during Aggregation to Be Delivered into Adjoining Cells

Next, these aggregates were used for interaction with MSCs to get insights into the mechanisms by which Fe-PEI aggregates may release their content inside the cells. To this aim, the interaction between MSCs and Fe-PEI aggregates was evaluated in two experimental settings: in the first case, the aggregates were allowed to assembly on tissue culture-treated 4-well plates (with cover glass surface) in serum-free medium in the presence of DiI for 24 h. After this time interval, the wells were washed thoroughly to remove any unbound fluorescent molecule and used for culturing MSCs. In the second experimental setting, the aggregates were assembled for 24 h in ULA plates in the presence of DiI, then removed, washed by centrifugation, resuspended in fresh complete culture medium and added onto pre-adhered MSCs cells. After 24 h of direct contact, the cells were examined under confocal microscope to evaluate the transfer efficiency of DiI from the aggregates into the cells.

**Figure 3:**
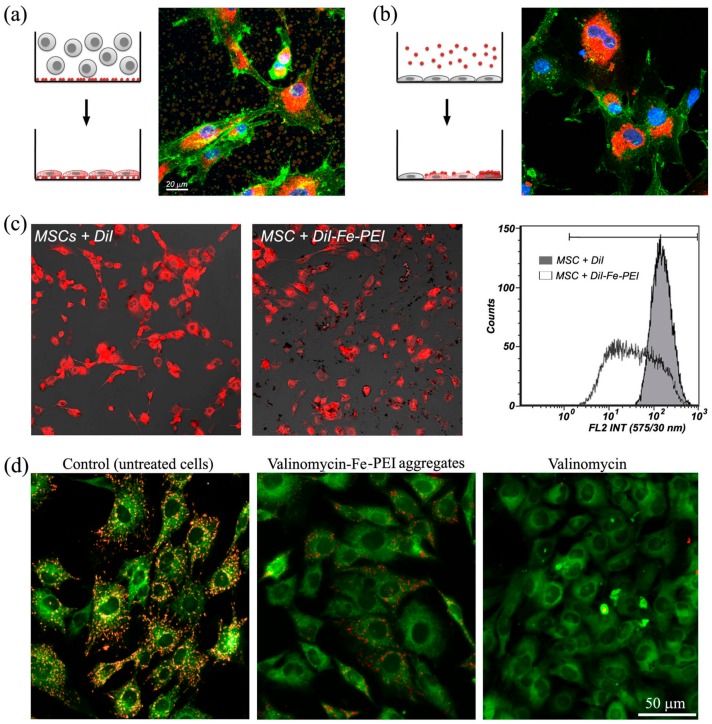
Delivery efficacy of molecules into MSCs using Fe-PEI. (**a**,**b**) Confocal microscopy images illustrating MSCs (actin staining with Oregon Green-labelled WGA) stained with DiI (red) after 24 h of direct contact between DiI-Fe-PEI aggregates with MSCs. The direct contact was achieved by seeding unstained MSCs on tissue culture plates coated with DiI-Fe-PEI aggregates (**a**) or by adding DiI-Fe-PEI aggregates into the culture medium of pre-adhered cells (**b**). Note that every single cell was fluorescent when allowed to adhere on the uniformly distributed aggregates (**a**); in contrast, when aggregate suspension was introduced into culture medium on the top of already adhered cells, only cells that established direct contact with DiI-Fe-PEI aggregates incorporated fluorochromes (**b**); (**c**) Quantification of red fluorescence in MSCs after interaction with DiI-Fe-PEI aggregates. (**d**) Representative picture of MSCs stained with JC-1 (red-orange for aggregate form of JC-1 and green for monomeric form) before (**left** image) and after exposure to valinomycin as such (**right** image), or valinomycin incorporated into Fe-PEI aggregates (**middle** image). Intact mitochondria appear as punctuate red fluorescence whereas depolarized mitochondria exhibit the characteristic diffuse green monomer fluorescence.

As illustrated in Figure 3a, when cell suspension was added onto aggregate-coated plates, all cells were uniformly stained with DiI, demonstrating an effective transfer of molecule by direct contact. On the contrary, when aggregate suspension was introduced into culture medium on the top of already adhered cells, only cells that established direct contact with DiI-Fe-PEI aggregates stained fluorescently (Figure 3b), while the others remained unstained. The lack of fluorescence in several cells further denoted the absence of DiI leakage from Fe-PEI aggregates and the specific transfer of fluorescent molecule strictly mediated by the direct contact between Fe-PEI aggregates and cells. Quantification of red fluorescence in MSCs after interaction with DiI-Fe-PEI aggregates in the second experimental setting described above was done by FACS analysis, in comparison to MSCs incubated with DiI-containing medium (Figure 3c). Although the presence of DiI molecule in the culture medium of MSCs induced a uniform fluorescent labeling of all cells (illustrated as the narrow peak with high fluorescence in FACS histogram), interaction of DiI-Fe-PEI aggregates with MSCs led to a broad spectrum of fluorescence in individual cells (illustrated as a larger peak with fluorescence varying from low to high). Together, these experiments confirmed that the transfer of DiI molecule from the aggregates to MSCs occurred only by direct contact and was correlated to the number of aggregates interacted with each individual cell.

Next, the capacity of Fe-PEI NPs to incorporate DiI by aggregation and subsequently release it into the cells they interact with was studied by using valinomycin, a lipid-soluble potassium ionophore able to induce mitochondrial membrane permeabilization and electrochemical potential dissipation. Valinomycin is known as a cargo that easily interacts and internalizes into cells. The effectiveness of valinomycin entrapped into Fe-PEI aggregates was assessed by evaluation of the mitochondrial membrane potential (ΔΨm) in MSCs after cell interaction with valinomycin-Fe-PEI aggregates using JC-1 [26]. As revealed in Figure 3d, ΔΨm was almost completely lost after 24-h exposure of MSCs to valinomycin-Fe-PEI aggregates, to a similar extent to MSCs exposed to the same dose of valinomycin given as such.

Taken together, this data demonstrates the capacity of Fe-PEI NPs aggregates to incorporate DiI and valinomycin in serum-free medium and deliver them into target cells by direct contact.

### 2.4. Fe-PEI NPs Increase the Efficiency of Intracellular Delivery of Cisplatin Incorporated during NPsSynthesis in Hydrothermal Conditions

Taking advantage of the mild conditions used in the hydrothermal synthesis of Fe-PEI NPs, as well as the strong adhesion of NPs to the cells, the capacity of Fe-PEI NPs to be used as a platform for drug delivery by incorporating anti-tumor drug cisplatin during synthesis process (Cis-Fe-PEI NPs) was evaluated. Due to the restrictions imposed by the solubility of cisplatin in water, the final concentration of cisplatin in NPs was 10 µM. Cis-Fe-PEI NPs retained similar characteristics and thermal properties as naked Fe-PEI NPs (Table A1 and data not shown).

**Figure 4:**
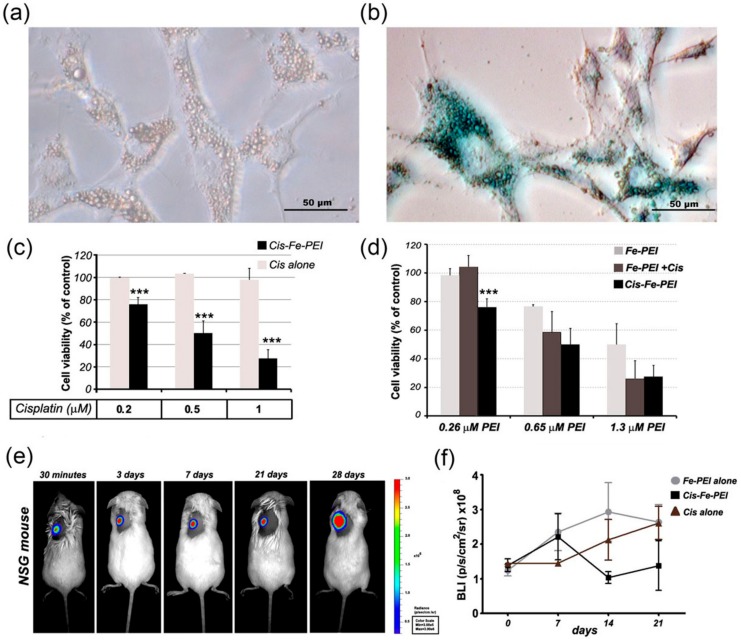
Increased efficiency of cisplatin drug on tumor cell death upon incorporation into Fe-PEI NPs during synthesis process. (**a**) Optical microscopy image showing the specific accumulation of Fe-PEI NPs onto tumor cell surface; (**b**) Optical microscopy image illustrating the appearance of blue aggregates on U87 cells after 24-h co-culture with Fe-PEI NPs; (**c**) Diagram illustrating U87 viability as determined by luciferase activity measured with IVIS after three days of culture in the presence of cisplatin alone or cisplatin incorporated into Fe-PEI during assembling process (Cis-Fe-PEI); (**d**) Diagram illustrating U87 cell viability (determined by luciferase activity, as in (**b**) in the presence of Fe-PEI, Cis-Fe-PEI (Cis added during Fe-PEI assembling), and Fe-PEI+Cis (cisplatin added post-synthesis), at various dilutions (equivalent of 0.26, 0.65, and 1.3 µM PEI, respectively); (**e**) Time-dependent tumor development after in vivo injection of 2 × 10^6^ cells into NSG mouse; (**f**) Initial tumor development in mice injected with U87 cells in the presence of 50 µL Cis-Fe-PEI (containing 0.5 µM Cis) applied at weekly intervals.

**Figure A4:**
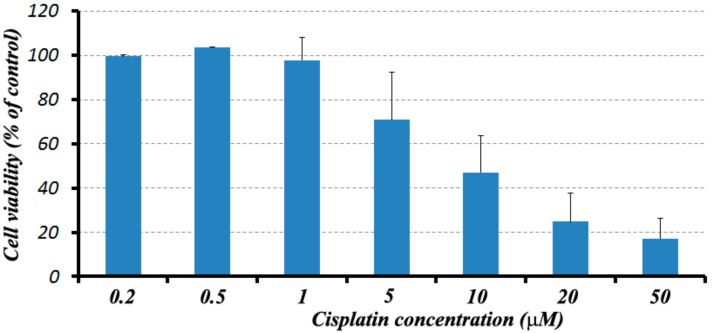
Cell viability of U87 in the presence of increasing concentrations of Cisplatin. Values were obtained as bioluminescent signal expressed as radiance in photons per second per square centimeter per steradian (p/s/cm^2^/sr) and expressed as percentage of control cells (treated in complete medium, in the absence of cisplatin).

The effectiveness of Cis-Fe-PEI NPs was assessed on luciferase expressing U87 glioblastoma cells. First, adhesive aggregates, similar to those formed by naked Fe-PEI NPs in contact with MSCs, were noticed after interaction of U87 cells with Cis-Fe-PEI NPs (Figure 4a), and the iron content for the cisplatin containing sample was evidenced by Prussian Blue staining (Figure 4b). Next, the outcome of cellular uptake of cisplatin was estimated through cell viability measured with an imaging system (Figure A4). To this aim, cells were allowed to adhere on tissue culture plates for 24 h before incubation with Cis-Fe-PEI, and the viability was determined 48 h later, based on luciferase signal. The survival curve of U87 cells in the presence of increasing concentrations of Cisplatin (ranging from 0.2 to 50 µM) showed that at concentrations less than 1 µM Cisplatin, U87 viability and proliferation were not affected (Figure A3). It was only in the presence of 5 µM or above when viability of U87 decreased. However, when 0.2, 0.5 and 1 µM Cisplatin embedded into Fe-PEI NPs were added onto U87 cells, their viability decreased to 76% (±5.9%), 50% (±11.2%) and 28% (±7.7%), respectively (Figure 4c). These results showed that cisplatin was much more effective in inhibiting tumor cell proliferation when administered as Cis-Fe-PEI NPs than when administered alone in equivalent doses.

Given that the cytotoxic effect of Cis-Fe-PEI NPs on U87 cells may partially be attributed to Fe-PEI NPs itself at higher concentrations, the impact of Cis-Fe-PEI was subsequently evaluated on U87 cells in comparison to equivalent amounts of naked Fe-PEI NPs and a mixture of naked Fe-PEI NPs and cisplatin (in equivalent doses). The results showed that, indeed, naked carrier Fe-PEI imparted cytotoxicity when present in high concentrations (more than 0.5 µM) in a dose-dependent manner (Figure 4d). However, for Fe-PEI NPs doses at which the viability of U87 cells was not affected, only Cis-Fe-PEI, but not Fe-PEI+Cis, significantly decreased cell viability (Figure 4d). This data confirmed that Fe-PEI NPs increased the efficiency of intracellular delivery of cisplatin when the latter was incorporated during hydrothermal synthesis process.

**Figure A5:**
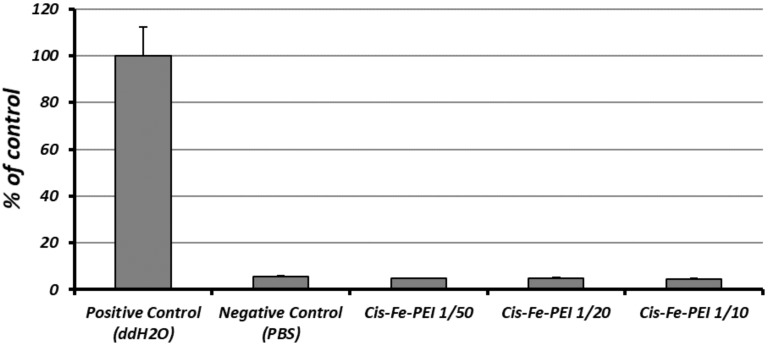
Haemolysis test revealing the hemocompatibility of Cis-Fe-PEI.

Finally, the effect of Cis-Fe-PEI was evaluated in vivo for its ability to limit the tumor formation and growth on a murine model bearing a heterotopic brain tumor. NSG (**N**od-**S**CID **G**amma-immunodeficient) mice were used, which lacked mature T cells, B cells, or functional NK cells, and were deficient in cytokine signaling, leading to better engraftment of cells than any other mouse strain. First, hemolysis test was performed and the NPs was proved to be haemocompatible (Figure A5). Next, tumor was induced subcutaneously by injection of 2 × 10^6^ U87 cells, and two days later the first dose of NPs was administered. No signs of inflammation were observed at the surgical site and no changes in body weight were observed before or after tumor induction or NPs implantation, suggesting that NPs were biocompatible with no associated toxicity or side effects.

**Figure A6:**
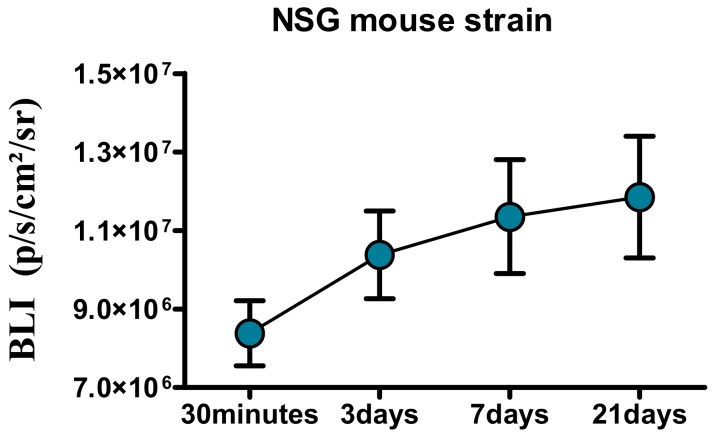
Tumor development in mice injected with U87 cells.

The impact of locally administered NPs (naked Fe-PEI, containing 0.65 µM NPs, or Cis-Fe-PEI, containing the same number of NPs and 0.5 µM cisplatin) on cancer cells was evaluated by bioluminescence imaging of mice using in vivo imaging system. As also reported by other groups [27], we noticed that the tumor started growing significantly only after 21 days after cell injection (Figure 4e and Figure A6). The results showed that administration of 50 µL Cis-Fe-PEI NPs (containing a very small dose of cisplatin, corresponding to 4 ng cisplatin/kg body weight, which is 10^5^ times lower than the dose usually used in vivo for therapeutic purposes) showed a tendency to reduce the onset of tumor progression at early time points in vivo (Figure 4f). Despite the fact that the statistical significance was not reached and the effect was transient, these data are encouraging for therapeutic purposes, as they demonstrated that anti-tumor drug cisplatin is more effective for therapeutic purposes when incorporated into Fe-PEI NPs than it is when administered by itself. Although we consider these studies encouraging, we are aware that further studies are necessary to validate the clinical efficiency of Fe-PEI NPs by assessing the effectiveness of Fe-PEI incorporating a clinically relevant dose of cisplatin on tumor reduction in vivo.

By concluding, the results presented here demonstrate that the incorporation of cisplatin into Fe-PEI NPs during hydrothermal synthesis is associated with the preservation of the biological activity of the molecule that can be further transferred into the target cells by direct contact. Such an approach might be valuable as a drug delivery strategy for solid tumor treatment.

## 3. Discussions

Various cationic polymers have been widely used as nanoscale systems to promote cellular uptake through the electrostatic interaction between their positive charge and the negative surface on the cell membrane. Among them, PEI has been reported to form cationic complexes with high uptake efficiency into the target cells either through passive transport or active endocytosis [18]. Several groups have reported various methods of preparation of Fe-PEI particles for diverse applications, including as MRI contrast agents, drug-delivery systems, or gene transfer, with minimal toxicity for the cells [28,29,30]. Furthermore, their magnetic properties were exploited in other newly emerging fields, such as biodetection [11] and hyperthermia [31,32,33] for non-invasive ways to target and treat cancer.

In the present work, we characterized the biological properties of Fe-PEI NPs obtained through hydrothermal synthesis [10] by following their fate in the culture medium, in the presence and absence of cells, as well as their ability to incorporate various biologically active factors (anti-cancer agent cisplatin, valinomycin and fluorescent dye DiI). The results presented here demonstrate that Fe-PEI NPs may incorporate the active molecules in the presence of culture medium and deliver them into MSCs by direct contact.

The mild conditions used in the hydrothermal synthesis of Fe-PEI NPs, as well as the strong adhesion of NPs to the cells, allowed us to introduce the anti-tumor drug cisplatin during synthesis process in order to assess the value of Fe-PEI NPs as platform for drug delivery. Cisplatin is a hydrophobic drug used in clinics for the treatment of a variety of solid tumors, such as glioblastoma, metastatic breast, ovarian and testicular cancers, osteogenic sarcoma, and neuroblastoma [34]. The substance becomes active upon entering the cell where it binds to DNA, inhibiting mitosis and inducing an apoptotic response [35]. The administration of cisplatin is frequently associated with considerable side effects like hepatotoxicity, cardiotoxicity and nephrotoxicity [35], hence a therapeutic approach that minimizes the side effects through a reduction in cisplatin dose while ensuring a sufficient cellular uptake for the drug action, appears to be a feasible strategy.

It is worth noting that the same concentration of Fe-PEI NPs thatcaused significant toxicity on the brain-derived glioblastoma cells resulted in no toxicity on MSCs. We therefore consider that these NPs could be also suitable for use as a drug-delivery platform in cell regeneration therapy, where they serve as carriers for the intracellular delivery of bioactive molecules (including small chemicals and drugs) to enhance stem cell potency with minimal adverse effects on their properties. Furthermore, the combination of this technique with anticancer drug-loaded NPs-MSCs could be a promising method of targeted delivery of MSCs to tumors. 

## 4. Materials and Methods

### 4.1. Preparation of Iron Oxide-PEI (Fe-PEI) Nanopowders

Hybrid nanostructures were prepared by hydrothermal synthesis in aqueous solution starting from FeCl_3_·6H_2_O, 25% ammonia solution, commercial PEI (Sigma Aldrich, St. Louis, MO, USA, average MW ~ 25,000 Da), as previously described [10]. Briefly, iron (III) chloride hexahydrate was mixed with ammonia solution to produce a strong alkaline suspension (pH = 10), which was then extensively washed until physiological pH, before being added to PEI (10% solution in water). The mixture was then transferred in stainless steel vessel of a closed autoclave endorsed with cooling system, for hydrothermal reaction at 40 °C and 100 atm. Pressure was created inside the reaction system using argon gas. The resulted hybrid nanostructures (herein referred to as Fe-PEI NPs) were lyophilized at −50 °C using a Martin Christ Alpha 1-2 LD Plus freeze dryer until Fe-PEI nanopowders were obtained.

### 4.2. Preparation of Fe-PEI Stable Aqueous Suspensions

Fe-PEI nanopowders obtained as described above were dispersed in different solutions in order to obtain stable aqueous suspensions to be used in biocompatibility assays. The following solutions have been tested: (i) 0.001 M sodium chloride solution (analytic reagent, Chimactiv, Bucharest, Romania) containing 50% (wt) poly(acrylic acid) PAA (electronic grade, Sigma-Aldrich, St. Louis, MO, USA), physiological pH (achieved by addition of 2 M NaOH solution, under continuous stirring) and stabilized by 30-min sonication (Elmasonic S10 (H) ultrasonic unit); (ii) phosphate buffer solution (PBS), physiological pH.

### 4.3. Incorporation of Cisplatin in Fe-PEI NPs

To evaluate the capacity of Fe-PEI NPs to incorporate drugs, cisplatin (cisdiamminedichloridoplatinum (II)) was added either before or after hydrothermal synthesis. In the case of cisplatin incorporation before hydrothermal synthesis (samples denoted as Cis-Fe-PEI), the solution containing drug was added in aqueous suspension of Fe-PEI mixture and the resulted suspension was transferred into the autoclave vessel for hydrothermal synthesis. During hydrothermal reactions, cisplatin likely reacted with iron oxide and PEI, resulting new physical bonds. In the case of cisplatin incorporated after hydrothermal synthesis (samples denoted as Fe-PEI+Cis), the drug was added to the newly formed Fe-PEI NPs immediately after hydrothermal synthesis. Thus, Fe-PEI+Cis is just a mixture of Fe-PEI NPs with cisplatin solution at room temperature, obtained by magnetic stirring, without any physical or chemical bonds between Fe-PEI NPs and cisplatin. Acronyms of investigated samples and experimental conditions in which they were obtained are summarized in Table 1.

### 4.4. Characterization of Fe-PEI Nanopowders

Synthesized nanostructured powders (both Fe-PEI hybrids and iron oxide NPs) were characterized by various analytical techniques. Chemical structure of the hybrids was determined using FT-IR spectroscopy. FT-IR spectra were recorded in the transmission mode from powders dispersed in KBr pellet, using a MB 3000 spectrometer (ABB, Montreal, QC, Canada) between 4000 and 550 cm^−1^ wavenumbers, with a resolution of 4 cm^−1^. HorizonMB software was used for automatic data processing. Thermogravimetric (DSC-TG) analysis of Fe-PEI sample was performed using a Setsys Evolution instrument (Setaram Instrumentation, Caluire, France). Data analysis was performed with Calisto software. Approximately 10 mg of each sample was examined in the temperature range 20–600 °C, in alumina crucible. The heating rate was 10°·min^−1^. Argon was used as carrier gas at a flow rate of 1 L·h^−1^ during TGA investigation. Particle size measurements were performed using Zetasizer ZS90 laser granulometer with zetapotential-(Malvern Instruments, Malvern, UK) with domain 0.6 nm–3.0 µm and specialized software. HRTEM (High Resolution Transmission Electron Microscope) analysis was performed using a Tecnai G2 F30 with S-Twin objective lens (FEI Company, Maastricht, The Netherlands). To this aim, samples were dispersed in ethylic alcohol to obtain suspensions, which were subsequently dropped on TEM copper grids coated with thin amorphous carbon film with holes.

### 4.5. Cells

Primary cultures of mouse bone marrow-derived mesenchymal stem cells (MSCs) and human umbilical cord-derived endothelial progenitor cells (EPCs), as well as a human glioblastoma cell line (U87-Luc cells from Perkin Elmer, Kristiansand, Norway) were used. Cells were grown in culture as previously described [36,37,38] or as indicated by manufacturer.

### 4.6. Prussian Blue Staining for Iron

The staining process is based on the chelation of iron by ferrocyanide, forming ferric ferrocyanide, which is blue in color. Briefly, cells grown on slides were fixed with 4% paraformaldehyde (PFA) and treated with a mixture of 20% hydrochloric acid: 10% potassium ferrocyanide solution (1:1) for 20 min. Slides rinsed with PBS were examined under light microscope.

### 4.7. The Interaction of Fluorescently-Labelled Fe-PEI NPs with MSCs

Fe-PEI NPs were allowed to aggregate for 24 h in DMEM in the presence of the fluorescent molecule DiI (1′-Dioctadecyl-3,3,3′,3′-Tetramethylindocarbocyanine Perchlorate Molecular Probes D-282, 30 µg/mL) either on ultra-low adherence (ULA) plates (that do not allow the adhesion of aggregates) or onto cell culture-treated chamber slides (in adherent conditions). Then, the aggregates were washed to remove not-entrapped fluorescent molecules and further used for the interaction with cells. To this aim, MSC suspension was seeded (at a density of 10,000 cells/cm^2^) into cell culture chamber slides (Lab-Tek II CC2 chamber slide system, Nunc, Rochester, NY, USA) either directly onto adhered Fe-PEI aggregates or in the presence of the suspension of Fe-PEI aggregates pre-formed in ULA conditions. The incorporation of DiI into MSCs was evaluated after 24 h. Briefly, cells were fixed with 4% PFA for 10 min at room temperature, followed by staining with Oregon Green-labelled WGA conjugate (wheat germ agglutinin, Molecular Probes, Eugene, OR, USA, 1/200 dilution) and Hoechst 33,258, for 10 min at room temperature. Washed slides were mounted with Prolong antifade reagent and examined by confocal microscopy (TCS-SP5 from Leica).

### 4.8. Bioluminescence Imaging on Cells in Culture

Taken advantage of the high expression of luciferase in U87 cells, the bioluminescent signal produced by these cells was used to determine cell viability after interaction with Fe-PEI NPs. To this aim, U87 cells were seeded in 96-well plates and allowed to grow for 24 h before being exposed to Fe-PEI NPs at various dilutions (equivalent to 110, 330 and 660 µg PEI/ml) for 3 days. At the time of cell viability analysis, cells were incubated with 150 µg/mLuciferin, followed by imaging on the IVIS Spectrum CT system (PerkinElmer, Kristiansand, Norway). Cell viability was expressed as percentage of bioluminescent signal produced by control cells.

### 4.9. Transmission Electron Microscopy (TEM)

Aggregates of Fe-PEI generated in DMEM were characterized by TEM. To this aim, 5 µL of suspension containing aggregated NPs were layered on a carbonated copper grid and left to dry. Then, the grid was counterstained with phosphotungstic acid 1% for 30 s and briefly washed in distilled water. At the end, the grid was allowed to dry and examined under the TEM (TECNAI Spirit Biotwin, FEI Company, Maastricht, The Netherlands).

### 4.10. Measurement of Mitochondrial Membrane Potential (ΔΨm)

This was assessed using the lipophilic cationic probe JC-1 (Invitrogen, Paisley, UK) that exhibits potential-dependent accumulation in mitochondria. This method is based on the ability of JC-1 to form red fluorescent aggregates when accumulated in normal mitochondria (with intact membrane potential). Loss of ΔΨm generates reduction of red fluorescence and a concomitant increase in green fluorescence (monomeric state of JC-1). After exposure of MSCs to Fe-PEI aggregates that entrapped valinomycin during 24-h aggregation in the culture medium, the cells were incubated with 5 µg/mL JC-1 for 10 min at room temperature in the dark. After washing with PBS, the cells were photographed using an epifluorescence microscope (Leica DMI6000, Wetzlar, Germany).

### 4.11. In Vivo U87 Cell Implantation

NSG mice were housed and used in accordance to national and European Union regulations for animal experimentation (Directive 2010/63/EU of the European Parliament) and all the procedures were approved by the Ethical Committee of the Institute of Cellular Biology and Pathology “N. Simionescu”. Tumors were induced in mice by subcutaneously injection of 2 × 10^6^ U87 in 50 µL DMEM into the interscapular region. Two days later, the mice received an intratumoral injection of 50 µL Fe-PEI NPs with cisplatin (*n* = 5), Fe-PEI NPs without cisplatin (50 µL control Fe-PEI, *n* = 5) or cisplatin alone (50 µL, *n* = 2). The injections were repeated three times, at weekly intervals. The concentration of cisplatin in the Fe-PEI suspension and cisplatin solution was 500 nM. Tumor development was recorded for up to one-month post-injection by in vivo imaging system, as previously described [38]. Details can be found in Appendix A.

## 5. Conclusions

Our data showed that the use of hydrothermal conditions for Fe-PEI NPs assembly allowed the incorporation of cisplatin, which remained therapeutically effective upon direct interaction with the cells. In addition, our data demonstrated that anti-tumor drug cisplatin was much more effective for inhibiting tumor cell proliferation, both in vitro and in vivo, when incorporated into Fe-PEI NPs than when administered alone in equivalent doses. Such an approach might be valuable as a drug delivery strategy for solid tumor treatment.

## Figures and Tables

**Table 1:** Synthesis parameters for nanostructured hybrids based on iron oxide-PEI.

Sample Name	Iron Oxide:PEI Mass Ratio	CisplatinPresence
Fe-PEI	0.5	no
Cis-Fe-PEI	0.5	Incorporated before hydrothermal synthesis
Fe-PEI+Cis	0.5	Incorporated after hydrothermal synthesis

## Supplementary Materials

The following are available online at http://www.mdpi.com/2079-4991/7/10/314/s1, Video Movie 1.

## Abbreviations

Cis	Cisplatin
Cis-Fe-PEI	poly(ethylenimine) (PEI)-coated iron oxide nanoparticles with cisplatin incorporated during synthesis
DiI	fluorescent molecule-1′-Dioctadecyl-3,3,3′,3′-Tetramethylindocarbocyanine Perchlorate
DiI-Fe-PEI	poly(ethylenimine) (PEI)-coated iron oxide nanoparticle aggregates incorporating DiI
DMEM	Dulbecco’s Modified Eagle Medium
EPC	endothelial progenitor cells
Fe-PEI NPs	poly(ethylenimine) (PEI)-coated iron oxide nanoparticles
Fe-PEI+Cis	poly(ethylenimine) (PEI)-coated iron oxide nanoparticles with cisplatin added post-synthesis
MSCs	mesenchymal stem cells
NP	nanoparticle
PAA	poly(acrylic acid)
PBS	phosphate buffer saline
U87	human glioblastoma cell line U87 (Luciferase positive cells)

## Appendix A

***Determination of size distribution with Nanosizer.*** The size distribution of Fe-PEI after aggregation in the presence of culture medium was determined with Nicomp 380 analyzer (Port Richey, FL, USA). The main parameters used were: liquid viscosity 1.05 cP, refractive index 1.33, intensity set point 300 kHz, first channel used 2, channel width set automatically and solid particle option for the analysis.

***xCELLigence analysis.*** *x*CELLigence analysis was performed to evaluate the biocompatibility of FePs, by real-time monitoring of cell behavior following the interaction with NPs. This system monitors cellular events in real time by measuring electrical impedance across interdigitated micro-electrodes integrated on the bottom of specially designed tissue culture E-plates. To investigate the effects NPs on EPC proliferation, NPs were added onto cell culture 24 h after EPCs adhered onto substrate. Briefly, EPCs are cultured on E-plates in endothelial growth medium (EGM-2, from Lonza-Basel, Switzerland) and 24 h later, once all cells had adhered to the substrate, the medium was replaced with fresh medium containing NPs (equivalent of 330 µg PEI/mL). EPCs incubated in EGM-2 in the absence of NPs are also included as controls.

***Haemolysis test.*** Mouse blood samples were collected on EDTA (Sigma-Aldrich, St. Louis, MO, USA) and erythrocytes were isolated by centrifugation at 1000 *g*, 4 °C, 15 min. The pellet was washed once with PBS and then re-suspended in the same volume of PSB they originated from. Aliquots of 100 µL of erythrocyte suspension were distributed in Eppendorf tubes and centrifuged again to pellet the erythrocytes. Each pellet was then re-suspended in 100 µL PBS containing Cis-Fe-PEI NPs (with 13 µM PEI and 10 µM cisplatin) at various dilutions (1/10, 1/20, 1/50) and incubated at 37 °C for one hour. A negative and positive control were run in parallel, by using distilled water and PBS, respectively. At the end, samples were centrifuged again and the supernatants were spectrophotometrically evaluated at 540 nm by using a TECAN Infinite M200Pro spectrophotometer (Mannedorf, Switzerland). All samples were reported as percentage of positive control, which was considered 100% haemolyzed.

In vivo ***U87 cell implantation***. Tumor development was recorded for up to one-month post-injection by in vivo imaging system. Briefly, anesthetized mice (with isoflurane) were intraperitoneally injected with luciferin (150 mg/Kg body weight) and 10 min later, they were imaged in dorsal position with IVIS Spectrum system (Perkin Elmer, Kristiansand, Norway). The following settings were used: field of view 6.6; binning factor 4; F-stop 2; exposure 15 s. Surface images were then analyzed using Living Image 4.3.1 software (PerkinElmer, Kristiansand, Norway) and quantification of bioluminescence was performed by manually defining regions of interest and reported as photons/second/square centimeter/steradian.

**Table A1:** Thermal properties of Fe-PEI nanostructured powders.

Sample	Peak 1, °C	ΔH, J/g	Δm, %	Peak 2, °C	ΔH, J/g	m, %	Peak 3, °C	ΔH, J/g	Δm, %	ΔmTotal, %
Fe-PEI	74.6	60	−8	390.5	34	30	547	179.5	−12.7	−71.7
Cis-Fe-PEI	74.9	49.5	−8	362.6	37.4	25	567	166	−14.6	−66
